# Association between Serum Cotinine Levels and Bone Mineral Density: An Analysis of the National Health and Nutrition Examination Survey (NHANES)

**DOI:** 10.1155/2022/6830705

**Published:** 2022-09-06

**Authors:** Jian-Guo Fang, Duo-Jun Wang, Hao-Yu Yang, Hui Zhang, Jin-Yu Tong, Zai-Jun Lin

**Affiliations:** ^1^Department of Spine Surgery, Shidong Hospital Affiliated to University of Shanghai for Science and Technology, No. 999, Shiguang Road, Shanghai 200438, China; ^2^School of Basic Medicine, Xinxiang Medical University, Xinxiang, Henan, China

## Abstract

**Purpose:**

To investigate the relationship between serum cotinine and lumbar bone mineral density (BMD) among 7905 participants aged 30 years and over.

**Method:**

A total of 3945 men and 3960 women from the National Health and Nutrition Examination Survey 2011–2018 were included in this cross-sectional analysis. Independent variable was serum cotinine, which is a biomarker of cigarette exposure. The outcome variable was lumbar BMD. We investigated the associations of serum cotinine levels and lumbar BMD using multivariable linear regression models.

**Results:**

Serum cotinine concentration was negatively associated with lumbar BMD after adjustment of relevant covariables (*β* = −0.039, 95% CI: −0.078 to −0.014, *P* = 0.005). However, in the subgroup analysis stratified by gender, this negative association remained only in women (*β* = −0.072, 95% CI: −0.132 to −0.012, *P* = 0.019).

**Conclusion:**

Our study suggested that elevated serum cotinine level correlated with decreased lumbar BMD, especially in women. This finding indicated that reducing cigarette exposure and maintaining serum cotinine at a low level may be beneficial to bone health for adults.

## 1. Background

Osteoporosis is a systemic disorder characterized by weakness of microarchitectural integrity of bone tissue resulting in bone fragility and consequently increase fracture risk. Fragility fractures are associated with a high mortality and heavy economic burden. It is reported that the cost related to fragility fractures was estimated at €37 billion in the European Union annually, and it will grow continuously in the future [1].

It is well established that smoking is an independent risk factor of osteoporosis and fragility fractures [2–4]. Besides, accumulated evidence has also shown the increased risk of osteoporosis in secondhand smoke exposures [5, 6]. According to several basic scientific studies, nicotine contributed to negative effect on mesenchymal stem cell proliferation and differentiation [7–9]. Nevertheless, nicotine is unstable and rapidly metabolizes to many metabolites in the liver. Cotinine is the main metabolite of nicotine, which has 8 times longer half-life than nicotine (nicotine 2 h, cotinine 16 h) and has been widely used as a biomarker of tobacco exposures [10–13]. Cotinine is present in the blood and urine of smokers.

Previous study focused on the relationship between urine cotinine level and bone mineral density (BMD) in adult males [14], but with limited evidence on the association between serum cotinine level and BMD in American adults over 30 years. We aim to investigate the association between tobacco determined by serum cotinine and lumbar BMD.

## 2. Materials and Methods

### 2.1. Study Population

The data were obtained from the National Health and Nutrition Examination Survey (NHANES), which is a survey research program conducted by the National Center for Health Statistics (NCHS). The NHANES program was designed to collect data about the health and nutritional status of adults and children in the United States. The applied research approach of this study is mainly according to Lu et al. [15].

We combined four NHANES cycles (2011-2012, 2013-2014, 2015-2016, and 2017-2018) when lumbar BMD and serum cotinine data were available. A total of 18,461 men and women older than 30 years were included. After exclusion of 1772 participants without serum cotinine data, 8689 participants without lumbar BMD data, and 784 with missing values on covariates, 7905 subjects remained in the final analysis.

### 2.2. Study Variables

The exposure variable of this study was serum cotinine. Serum cotinine is measured by an isotope-dilution high-performance liquid chromatography/atmospheric pressure chemical ionization tandem mass spectrometric (ID HPLC-APCI MS/MS) method. The outcome variable was lumbar BMD. Lumbar BMD was measured by dual-energy X-ray absorptiometry (DXA). For covariates in this study, gender, race/ethnicity, education level, and physical activity were used as categorical variables; age, body mass index (BMI), serum albumin, blood urea nitrogen, serum uric acid, serum calcium, and serum phosphorus were used as continuous variables.

### 2.3. Statistical Analyses

We performed all statistical analyses by using statistical software R (Version 4.1.3). A two-tailed *P* value <0.05 was considered statistically signiﬁcant. Because of the complex survey design of NHANES, we calculated the new weight of our survey data according to the analytical guideline edited by NCHS. Baseline characteristics and serum cotinine concentration are presented as numbers and weighted percentages for categorical variables and as weighted mean ± standard error (SE) for continuous variables. We used the weighted two-tailed *t* tests for continuous variables or the weighted Rao-Scott chi-square test for categorical variables to calculate the diﬀerence among males and females. We constructed weighted multivariate linear regression models to evaluate the association of serum cotinine and lumbar BMD. Subgroup analyses stratified by sex and race were further performed. A weighted generalized additive model and a smooth curve fitting were conducted to address for non-linearity.

### 2.4. Statement of Ethics

The study was approved by the ethics review board of the National Center for Health Statistics, and written consent was obtained from each participant.

## 3. Result

A total of 7905 participants over 30 years were included in this study. The weighted distributions of the characteristics according to gender are shown in Table 1. Compared with men, women had higher levels of education, higher moderate recreational activities, higher BMI, and higher BMD, but lower levels of serum albumin, blood urea nitrogen, serum uric acid, and serum calcium, lower serum phosphorus, and lower serum cotinine.

The results of the multivariate regression analyses are presented in Table 2. In the unadjusted model, serum cotinine was negatively correlated to lumbar BMD (*β* = −0.037, 95% CI: −0.068 to −0.005, *P*=0.026). After adjustment for confounders, this negative association was still present in model 2 (*β* = −0.061, 95% CI: −0.092 to -0.029, *P* < 0.001) and model 3 (*β* = −0.039, 95% CI: −0.078 to −0.014, *P*=0.005). However, in the subgroup analyses stratified by gender and race, this negative association was still present only in women (*β* = −0.072, 95% CI: −0.132 to −0.012, *P*=0.019) and non-Hispanic black and non-Hispanic white groups.

**Figure 1:**
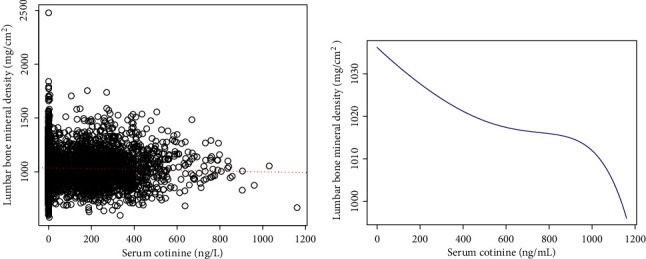
The association between serum cotinine and lumbar bone mineral density. (a) Each black point represents a sample. (b) Smooth curve fit between serum cotinine and lumbar bone mineral density. Age, gender, race/ethnicity, education, body mass index, physical activity, serum albumin, blood urea nitrogen, serum uric acid, serum calcium, and serum phosphorus were adjusted.

**Figure 2:**
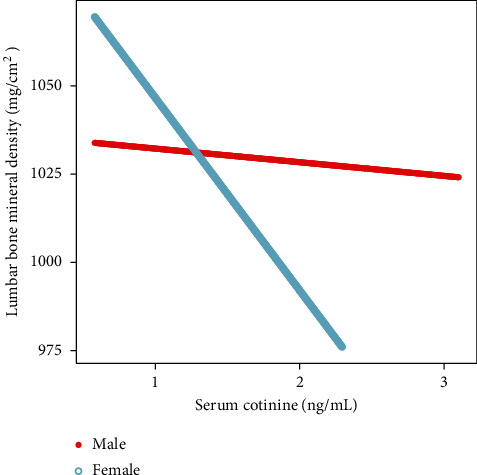
The association between serum cotinine and lumbar bone mineral density stratiﬁed by sex. Age, race/ethnicity, education, body mass index, physical activity, serum albumin, blood urea nitrogen, serum uric acid, serum calcium, and serum phosphorus were adjusted.

**Figure 3:**
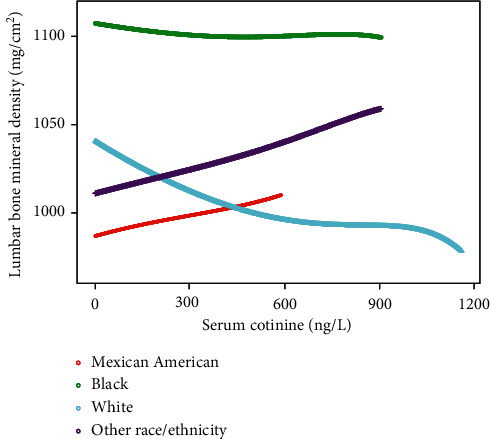
The association between serum cotinine and lumbar bone mineral density stratiﬁed by race/ethnicity. Age, gender, education, body mass index, physical activity, serum albumin, blood urea nitrogen, serum uric acid, serum calcium, and serum phosphorus were adjusted.

The associations between serum cotinine and lumbar BMD were further confirmed by generalized additive models and smooth curve fittings (Figures 1–3).

## 4. Discussion

To the best of our knowledge, this study is the largest population-based study to explore the association of serum cotinine and BMD in a nationally representative sample of US adults. In this cross-sectional survey, we found that elevated serum cotinine level correlated with decreased lumbar BMD, especially in women. Although we found some non-linear associations between them, the trends were consistent with our multivariable linear regressions.

It is estimated that over 150 molecular species have been identiﬁed as toxicants in cigarettes [16]. Also, the mechanism behind smoking and decreased BMD remains to be elucidated. Many studies used self-reported smoking to investigate the relationship between smoking and BMD [2, 5, 17, 18]. Nevertheless, compared to self-reported smoking rates, the true smoking rates are generally underestimated [19, 20]. Passive smoking is usually determined by self-report information or by measuring nicotine metabolites in body fluids such as urine, saliva, and blood. Self-report surveys are commonly used in studies assessing smoking prevalence because of their availability and economic feasibility. Serum cotinine measurement has been widely used to assess tobacco exposure [21, 22].

From our results, the serum cotinine level in men was nearly 2 times higher than women in the US civilian, non-institutionalized population. Also, serum cotinine was more closely associated with decreased lumbar BMD in female adults. Different from our findings, an NHANES III cross-sectional study showed association of serum cotinine and decreased BMD both in males and females [21]. It is suggested that serum cotinine concentration from our result was much lower. It indicated reducing cigarette exposure in 2011–2018 compared to 1988–1994 [21]. Furthermore, we observed that cotinine levels have 4 times greater negative impact on women than men. The potential mechanism remained unclear. However, several studies reported that smoking might have a direct negative effect on estrogen metabolism, resulting in consumption of estrogen and a subsequent decrease in bone mass [23, 24]. Gu et al. also found lower urinary levels of total estrogen metabolites in female smokers and confirmed an association between smoking and estrogen metabolism [25].

Previous studies demonstrated that nicotine exerts a dual-directional regulation on proliferation and diﬀerentiation of mesenchymal stem cells [7, 26, 27]. In vitro experiments demonstrated that no difference was observed in survival and proliferation of bone marrow-derived mesenchymal stem cells when they were exposed to nicotine at low concentrations (1 *μ*M to 100 *μ*M). However, cell proliferation decreased significantly in high concentration of nicotine (over 5 mM) [7]. Moreover, from our fitted curve, when serum cotinine level increases over 800 ng/L, lumbar BMD decreases markedly. Interestingly, our subgroup analyses stratiﬁed by race/ethnicity showed that Mexican Americans presented a positive association between serum cotinine and lumbar BMD although not significant. We particularly found that the mean cotinine level of Mexican Americans is 19.59 ± 63.58 ng/L, which is much lower than that of non-Hispanic black (86.38 ± 151.79 ng/L) and non-Hispanic white (99.02 ± 162.59 ng/L) groups. It means Mexican Americans experience less cigarette exposure. The low cigarette exposure in Mexican Americans might have minor effect on bone mass. Diﬀerences in genetic risk factors, BMI, alcohol nutrition intake, and other factors may provide a possible explanation for the race-speciﬁc diﬀerences. Restricted by the considerably smaller sample size and the nature of this cross-sectional study, the potential mechanism behind it is unclear. Further prospective studies need to be conducted.

Our study has several limitations. First, we conducted the fitted curve, but limited to the use of weight, we failed to calculate the specific breakpoint of the curve. Second, we did not take into account other confounding factors, so there is a possibility of bias. Third, studies with large sample sizes are needed to understand the association between serum cotinine and lumbar BMD.

## 5. Conclusion

Our findings suggest that tobacco exposure by active or passive smoking seems to exert a negative effect on lumbar BMD. This finding indicated that reducing cigarette exposure and maintaining serum cotinine at a low level may be beneficial to bone health for adults.

## Figures and Tables

**Table 1:** Weighted characteristics of study participants.

Characteristics	Men (*n* = 3945)	Women (*n* = 3960)	*P* value
Age (years)	45.03 ± 0.18	45.41 ± 0.19	0.069
Race/ethnicity (%)			0.004
Mexican American	10.33 ± 0.02	9.03 ± 0.01	
Non-Hispanic black	10.10 ± 0.01	12.03 ± 0.01	
Non-Hispanic white	63.52 ± 0.02	62.84 ± 0.02	
Other race/ethnicity	16.04 ± 0.01	16.18 ± 0.01	
Education level (%)			0.003
≤ high school	22.91 ± 0.01	18.85 ± 0.01	
＞ high school	77.09 ± 0.01	81.15 ± 0.01	
Body mass index (kg/m^2^)	29.45 ± 0.16	29.83 ± 0.21	0.088
Moderate activities (%)			<0.001
Yes	34.20 ± 0.01	14.63 ± 0.01	
No	65.80 ± 0.01	85.27 ± 0.01	
Serum albumin (g/L)	43.70 ± 0.01	41.85 ± 0.10	<0.001
Blood urea nitrogen (mg/dL)	5.02 ± 0.04	4.38 ± 0.04	<0.001
Serum uric acid (mg/dL)	6.03 ± 0.03	4.66 ± 0.02	<0.001
Serum phosphorus (mg/dL)	1.17 ± 0	1.12 ± 0	<0.001
Serum calcium (mg/dL)	9.38 ± 0.01	9.30 ± 0.01	<0.001
Lumbar BMD (mg/cm^2^)	1031.60 ± 3.27	1035.46 ± 3.72	<0.001
Serum cotinine (ng/L)	82.55 ± 4.40	49.65 ± 3.05	<0.001

Data are expressed as weighted proportions (± standard error (SE)) for categorical variables and as weighted mean ± SE for continuous variables. Weighted two-tailed *t* tests and weighted Rao-Scott chi-square test were used to compare difference between groups.

**Table 2:** The association between serum cotinine (ng/mL) and lumbar bone mineral density (mg/cm^2^).

	Model 1	Model 2	Model 3
	*β* (95% CI) *P* value	*β* (95% CI) *P* value	*β* (95% CI) *P* value
Serum cotinine (mg/dL)	−0.037 (−0.068, −0.005) 0.026	−0.061 (−0.092, −0.029) <0.001	−0.046 (−0.078, −0.014) 0.005
Serum cotinine categories			
Q1	Reference	Reference	Reference
Q2	−7.505 (−19.196, 4.187) 0.204	−9.279 (−20.975, 2.417) 0.118	−8.712 (−20.455, 3.019) 0.142
Q3	−4.270 (−16.707, 8.167) 0.495	−13.305 (−26.378, −0.232) 0.046	−13.238 (−26.281, −0.194) 0.047
Q4	−15.595 (−28.402, −2.787) 0.018	−26.983 (−40.266, −13.700) <0.001	−22.771 (−35.842, −9.700) 0.001
Subgroup analysis stratiﬁed by sex			
Men	−0.018 (−0.055, 0.020) 0.355	−0.040 (−0.079, − 0.002) 0.038	−0.020 (−0.059, 0.018) 0.297
Women	−0.072 (−0.132, −0.012) 0.019	−0.099 (−0.156, −0.042) <0.001	−0.089 (−0.146, −0.033) 0.002
Subgroup analysis stratiﬁed by race/ethnicity			
Mexican American	0.045 (−0.106, 0.196) 0.550	0.062 (−0.090, 0.214) 0.413	0.072 (−0.076, 0.220) 0.331
Non-Hispanic black	−0.018 (−0.084, 0.047) 0.573	−0.030 (−0.094, 0.034) 0.346	−0.013 (−0.075, 0.04) 0.667
Non-Hispanic white	−0.084 (−0.124, −0.043) <0.001	−0.082 (−0.123, −0.041) <0.001	−0.066 (−0.107, −0.061) 0.002
Other race/ethnicity	0.047 (−0.027, 0.121) 0.210	0.039 (−0.036, 0.115) 0.304	0.044 (−0.034, 0.121) 0.265

Model 1: no covariates were adjusted. Model 2: age, gender, and race/ethnicity were adjusted. Model 3: age, gender, race/ethnicity, education, body mass index, physical activity, serum albumin, blood urea nitrogen, serum uric acid, serum calcium, and serum phosphorus were adjusted. In the subgroup analysis stratiﬁed by sex and race/ethnicity, the model is not adjusted for sex and race/ethnicity, respectively.

## Data Availability

The survey data are publicly available on the Internet for data users and researchers throughout the world (www.cdc.gov/nchs/nhanes/).
